# Lung Cancer Screening with Low-Dose CT: What We Have Learned in Two Decades of ITALUNG and What Is Yet to Be Addressed

**DOI:** 10.3390/diagnostics13132197

**Published:** 2023-06-28

**Authors:** Mario Mascalchi, Giulia Picozzi, Donella Puliti, Stefano Diciotti, Annalisa Deliperi, Chiara Romei, Fabio Falaschi, Francesco Pistelli, Michela Grazzini, Letizia Vannucchi, Simonetta Bisanzi, Marco Zappa, Giuseppe Gorini, Francesca Maria Carozzi, Laura Carrozzi, Eugenio Paci

**Affiliations:** 1 Department of Clinical and Experimental Biomedical Sciences “Mario Serio”, University of Florence, 50121 Florence, Italy; 2 Division of Epidemiology and Clinical Governance, Institute for Cancer Research, Prevention and Clinical Network (ISPRO), 50100 Florence, Italy; g.picozzi@ispro.toscana.it (G.P.); d.puliti@ispro.toscana.it (D.P.); zappamarco@hotmail.com (M.Z.); g.gorini@ispro.toscana.it (G.G.); paci.eugenio@gmail.com (E.P.); 3 Department of Electrical, Electronic, and Information Engineering “Guglielmo Marconi”, University of Bologna, 47521 Cesena, Italy; stefano.diciotti@unibo.it; 4 Radiodiagnostic Unit 2, Department of Diagnostic Imaging, Cisanello University Hospital of Pisa, 56124 Pisa, Italy; a.deliperi@ao-pisa.toscana.it (A.D.); chiara.romei@gmail.com (C.R.); f.ba.falaschi@gmail.com (F.F.); 5 Pulmonary Unit, Cardiothoracic and Vascular Department, University Hospital of Pisa, 56124 Pisa, Italy; francesco.pistelli@unipi.it (F.P.); laura.carrozzi@unipi.it (L.C.); 6 Division of Pneumonology, San Jacopo Hospital Pistoia, 51100 Pistoia, Italy; michela.grazzini@uslcentro.toscana.it; 7 Division of Radiology, San Jacopo Hospital Pistoia, 51100 Pistoia, Italy; letizia.vannucchi@uslcentro.toscana.it; 8 Regional Laboratory of Cancer Prevention, Institute for Cancer Research, Prevention and Clinical Network (ISPRO), 50100 Florence, Italy; s.bisanzi@ispro.toscana.it (S.B.); frakaro@gmail.com (F.M.C.)

**Keywords:** biomarkers, coronary artery calcifications, emphysema, low-dose CT, lung cancer, lung nodules, mortality, radiations, screening, smoking

## Abstract

The ITALUNG trial started in 2004 and compared lung cancer (LC) and other-causes mortality in 55–69 years-aged smokers and ex-smokers who were randomized to four annual chest low-dose CT (LDCT) or usual care. ITALUNG showed a lower LC and cardiovascular mortality in the screened subjects after 13 years of follow-up, especially in women, and produced many ancillary studies. They included recruitment results of a population-based mimicking approach, development of software for computer-aided diagnosis (CAD) and lung nodules volumetry, LDCT assessment of pulmonary emphysema and coronary artery calcifications (CAC) and their relevance to long-term mortality, results of a smoking-cessation intervention, assessment of the radiations dose associated with screening LDCT, and the results of biomarkers assays. Moreover, ITALUNG data indicated that screen-detected LCs are mostly already present at baseline LDCT, can present as lung cancer associated with cystic airspaces, and can be multiple. However, several issues of LC screening are still unaddressed. They include the annual vs. biennial pace of LDCT, choice between opportunistic or population-based recruitment. and between uni or multi-centre screening, implementation of CAD-assisted reading, containment of false positive and negative LDCT results, incorporation of emphysema. and CAC quantification in models of personalized LC and mortality risk, validation of ultra-LDCT acquisitions, optimization of the smoking-cessation intervention. and prospective validation of the biomarkers.

## 1. Introduction

Lung cancer (LC) is among the most common and lethal neoplasms, accounting for about 2 million cases and 1.7 million deaths per year worldwide [1]. Accordingly, despite some improvement following molecular characterization and target therapy [2], the overall five-year survival rate of LC is just 20.5% [3]. The main histological types of LC include adenocarcinoma, squamous-cell carcinoma, and small-cell lung cancer. Screening with chest X-rays and sputum cytology were tested without efficacy [4,5,6,7]. 

Following pioneer experiences in Japan [8,9], in 1999, Claudia Henschke et al. published [10] the results of a trial named the Early Lung Cancer Action Program (ELCAP) in which screening chest low-dose computed tomography (LDCT) demonstrated a greater number of LC in earlier surgically curable stages than chest X-rays in subjects with relevant smoking history. Two years later, the ELCAP group reported the results of the next two annual LDCT rounds indicating lower overall positivity (2.5% vs. 23%) and LC detection (7/1184 = 0.0059 vs. 27/1000 = 0.027) rates as compared to baseline screening [11]. Based on these promising results, observational (one-arm) studies in which cohorts of smokers or former smokers underwent annual LDCT for many years were initiated in 12 centers in New York City (NY-ELCAP), in 82 centers worldwide (International-ELCAP) [12], and in Milan, Italy [13].

Since observational studies can suffer from biases, randomized controlled trials (RCTs) are considered as necessary to definitely demonstrate the efficacy of a health intervention and, specifically, the capability of LDCT to decrease LC mortality. Accordingly, in the US, after a small pilot, the lung-screening study (LSS), a RCT named the National Lung Screening Trial (NLST), was performed between 2002 and 2004 comparing LC mortality in 26,722 smokers or former smokers receiving three annual LDCT vs. 26,732 smokers or former smokers receiving three annual single view PA chest X-rays. The results of the trial demonstrating a relative LC mortality reduction of 20% in the LDCT arm were published in 2011 [14], and an 11-year extension of follow-up was available in 2019 [15].

Between 2001 and 2011, several small RCTs in subjects with relevant smoking history began in Europe, including Denmark [16], France [17], Germany [18] and, especially, Italy, where three studies were conducted, namely, the DANTE [19], MILD [20], and ITALUNG [21] trials. In the same period, a more powered trial, named NELSON, comparing annual or biennial LDCT with usual care, was initiated in The Netherlands [22]. Finally, a RCT offering a single LDCT vs. usual care was started in 2010 in UK [23].

An ad hoc committee of the European Union judged that the Danish, German, ITALUNG, and NELSON RCTs had low risk of biases and high quality of evidence [24]. The same trials plus the NLST and DANTE trials were considered as valuable sources for recommendations of LC screening by the United States Prevention Service Task Force (USPSTF) [25].

A variable mortality reduction in smokers or former smokers undergoing annual LDCT was observed in all European trials, except for the Danish and DANTE trials, and in the UK trial, and a meta-analysis of nine trials measured a relative risk of 0.84 of LC mortality (95% CI 0.76–0.92) [26]. The benefit of LC screening demonstrated by RCTs renders LDCT the cornerstone of today’s LC screening policy, along with smoking cessation, and in 2021 the USPSTF has recommended annual LDCT screening for LC in subjects aged 50 to 80 with a smoking history of at least 20 pack years, including current smokers and former smokers who had quit in the last 10 years only [27]. Although some studies in eastern countries reported a benefit of LDCT screening also in never-smokers [28,29], such an intervention is not recommended in western countries.

Herein, we recapitulate and critically summarize the work carried out and the scientific papers published within the frame of the ITALUNG trial whose pilot study was performed between 2001 and 2004 [30], namely, about two decades ago. The work covered several issues of the screening intervention in smokers and former smokers including design and recruitment, lung nodule detection and measurements, results of the LDCT, LC incidence, and mortality outcomes, smoking-related comorbidities, smoking cessation, radiations exposure, results and potential roles of biomarkers, and participation in production of position papers (Table 1). We strived to emphasize the contributions of the ITALUNG study to national and international LC screening activity.

## 2. The ITALUNG (Italy Lung Screening) Randomised Trial 

The study was conducted in accordance with the amended Declaration of Helsinki (http://www.wma.net/en/30publications/10policies/b3/, accessed on 2 January 2023) and was approved by the local ethic committees of the participating institutions (approval number 29–30 of 30 September 2003; number 23 of 23 October 2003; and number 00028543 of 13 May 2004)

### 2.1. Design

In the ITALUNG RCT, we compared an active arm undergoing LDCT screening and a control arm receiving usual care in which no intervention, in particular, chest X-rays, was offered. Moreover, we used a stop-screening design in which the evaluated intervention, i.e., annual LDCT, was offered to the active arm for a limited period of time, in our case, four years, while the LC incidence, and overall and disease-specific mortality data were collected in both the active and control arms for a longer period. This allows measuring the effects of the intervention on the primary screening objectives, namely, the overall and disease-specific (LC, cardio-vascular, and respiratory disease) mortality and the LC incidence. The possibility of contamination, namely, execution of LDCT in subjects allocated to the control arm, was unlikely at the time of active screening in ITALUNG (between 2004 and 2010) because the NLST results establishing the benefit of annual LDCT were published only in 2011. 

### 2.2. Recruitment

The risk of developing LC depends on a number of factors. They, basically, include age, smoking history, and exposure to environmental agents, and are variably reflected in the recruitment strategies of subjects to be screened. Schematically, recruitment can follow two paths: (1) self-referral of volunteers reached by advertising who can access toll-free phone numbers or website (opportunistic screening); and (2) population-based (organized) screening as it is implemented in many developed countries for breast, cervical, and colorectal cancer, in which subjects are actively invited to undergo screening by local public health institutions. Unfortunately, differently from other screening, in the case of LC, at least in Western countries, one must assess eligibility in terms of relevant smoking history before the invitation to screening intervention. This is particularly critical in the case of organized screening in which only at-risk subjects represent the intervention target to be invited. 

In ITALUNG, subjects aged between 55 and 69 years and living in the Florence, Pisa, or Pistoia districts, and identified through the list of subjects in charge at 269 general practitioners (GPs), received a mail invitation containing a questionnaire exploring eligibility that was defined as a smoking history of at least 20 packs/years (one pack for day for 20 years) and a less-than-10-years period of smoking quit in the case of former smokers. If eligible, they were centrally randomized using numbers generated by a computer to the LDCT screening test or the control arm. In ITALUNG, the multi-centre recruitment strategy with mail invitations to assess eligibility was associated with a very low yield of respondents and eligible subjects. In fact, to identify 3206 eligible subjects, we sent 71,232 letters with a yield of just 4.5% [21].

All eligible subjects were invited to attend the local centre for smoking cessation, if current smokers. Subjects randomized to the active group were invited to a face-to-face consultation with a pneumologist who assessed health conditions and provided detailed information about LDCT screening. The subjects of the control group received a letter signed by the study principal investigator inviting her/him to refer to the GP in case of onset of major respiratory symptoms.

Notably, in ITALUNG, we ascertained eligibility using simple age and smoking burden threshold criteria and observed a 19% lower yield of screen-detected LC than that obtained in the pilot UKLS trial that used a more articulated risk questionnaire comprising also evaluation of asbestos exposure, history of respiratory disease, or familial LC, that are included in the validated Liverpool Lung Project risk model (LLPv2) [42,61].

### 2.3. Structure 

ITALUNG was a multicentre study that involved three centres of active LDCT screening in the Tuscany region of Italy (Florence, Pistoia, and Pisa). Diagnostic workup was carried out locally, and this implied some variability in the diagnostic workup procedures that included one- or three-month follow-up LDCT, 18-Fluoro-Deoxy Glucose Positron Emission Tomography (FDG-PET), CT-guided fine-needle aspiration or core biopsy or video-assisted thoracic surgery (VATS) and bronchoscopy. Another major difference among the three centres was the efficacy of the accompanying invitation to a free smoking-cessation program that was offered to all randomized subjects, but attended mainly in the Pisa screening centre [53].

The primary outcomes of ITALUNG were centrally established with a link to the mortality and cancer registries of the Tuscany regions.

### 2.4. Nodule Detection and Measurements

The radiological protocol for LDCT acquisition and reading, the definition of positive screening tests, and the diagnostic workup were the same in the three screening centres and, substantially, matched those of the international ELCAP [21,38]. Eight CT scanners were used [55] between 2004 and 2010 to execute four screening rounds, and 17 board-certified radiologists with at least five years of experience in chest CT read the LDCT examinations [38]. Despite research work on in-house developed computer-assisted diagnosis (CAD) systems [31,32,36], a double reading of the LDCT test by independent radiologists was performed with consensus in case of disagreement, the same as in breast screening. 

Lung nodules size determines the screening-test result [59,62]. Aware of the greater sensitivity of volume as compared to diameters to measure nodule size and its changes over time [59], within the frame of ITALUNG, we developed and tested several algorithms to automatically or semi-automatically (after guided manual editing) measure nodule volume or characteristic scale [34,35,37]. However, the persistent 10-15% proportion of not properly segmented solid nodules [34,37] and the uncertain accuracy of software for volumetric assessment of non-solid or part-solid nodules [59] led us to prefer the use of electronic callipers to measure mean diameters of all solid, non-solid, and part-solid nodules detected in LDCT, well recognizing the implications in terms of imperfect reproducibility of this choice [33].

### 2.5. Results of LDCT

The results of the LDCT screening in ITALUNG, using a threshold of 5 mm in diameter for solid nodules and 10 mm for non-solid nodules at baseline, and 3 mm for solid nodules at annual repeat, were in line with other studies. We observed a 30.3% positivity at baseline and 15.8 % positivity at annual repeat and a rate of screen-detected LC of 1.5% within one year of baseline and of 0.5% in the three next years [21,38].

ITALUNG contributed to focus on three aspects of LDCT screening, namely, that most of the screen-detected LCs are already present at baseline LDCT, but can escape report because of the small size [39], that screen-detected LC can present as lung cancer associated with cystic airspaces (LCCA) [63,64], and that smokers and former smokers undergoing LDCT screening can develop multiple primary lung cancers [40,41].

In particular, a review of all the LDCT examinations of 20 cases of screen-detected LC in ITALUNG, which were diagnosed after the first annual repeat LDCT (and were initially considered “incident” LC) revealed that in 17 (85%) of them focal nodular or non-nodular lung abnormalities were already present at baseline LDCT in the site of the later diagnosed LC [39]. Since the early features of LC are not specific and are shared with benign nodules, while growth is a distinctive feature of nodule malignancy, the main implication of this observation is that all focal pulmonary abnormalities detected in screened subjects should be re-evaluated in subsequent LDCTs, especially for possible intervening size or density increase [39].

LCCA is a distinctive presentation of LC [63,65] which can occur also within the frame of LC screening, accounting for 2% of LCs identified at baseline LDCT and for 12% of LCs identified at annual repeat LDCT [66]. While LCCA is predominantly associated with adenocarcinoma, cases of squamous-cell carcinoma, carcinoid, non-differentiated carcinoma, and small-cell lung cancer presenting as LCCA were reported [64]. The closer follow-up possible in LDCT screening is expected to provide a more complete representation of the respective evolution of the non-solid, solid, and cystic components that are characteristic of LCCA, but whose variable combination renders difficult the application of software for volumetric size assessment of this type of LC [64]. 

Overall, 16 primary lung cancers (SPLC) occurred in six subjects of the active group of ITALUNG [41]: 12 LC in four subjects during the active screening and 4 LC in two subjects after the end of screening. These data confirm that the risk of SPLC is within the 1%-to-2% range per patient per year as defined in clinical series [40,67].

Moreover, considering the associated lung findings, we proposed a schematic operational classification of mediastinal lymphadenomegaly observed in screening LDCT examinations [68]. In the case of non-calcified lung nodules, detection of mediastinal lymphadenomegaly justifies a higher suspicion for nodule malignancy, and appropriate diagnostic workup on the associated nodule or enlarged lymph nodes is recommended (as recognized in Lung-RADS 1.1 classification, see below). However, mediastinal lymphadenopathy can also be observed in subjects with benign diffuse lung diseases (infectious, inflammatory, fibrotic, and granulomatous) or congestive heart failure that are easily demonstrated by LDCT, and, in such cases, the probability of malignant nature of the enlarged lymph nodes is low and conservative management is advised with follow-up LDCT. Finally, mediastinal lymphadenomegaly can be observed in the absence of any lung abnormality. While the possibility of a lymphoma or even metastases from extra-pulmonary malignancy must be entertained, in this scenario we suggest a careful re-evaluation of the lung and the airways to search for possibly overlooked LC.

### 2.6. Outcomes

Despite a LC mortality decrease of 30% after 9.3 years and of 24% after 11.3 years of follow-up [43,49] in subjects of ITALUNG undergoing LDCT, the differences with the LC mortality in the control arm did not reach statistical significance, presumably, because of the small sample sizes. The greater benefit of LDCT screening in women, as observed in the NLST, LUSI, and NELSON trials, was confirmed in ITALUNG, although, also in this case, the difference was not statistically significant [45].

Differently, the overall mortality after 11.3 years was significantly lower in subjects of the active arm (OR 0.80 with 95CI = 0.66–0.96) due to additive lower mortality for LC and for cardio-vascular disease (CVD) (see below). In particular, the analysis of the underlying factors of the decreased CVD indicated that inclusion of information about the presence of coronary artery calcifications (CAC) in the LDCT report might potentially be the explaining element for this difference, by promoting interventions of primary or secondary CV prevention in subjects with CAC [49].

Overdiagnosis, namely, the detection through screening of a cancer that would never have been identified in the lifetime, is an adverse outcome of screening [69]. We conducted a long-term survival analysis by prognostic categories and concluded against the long-term risk of overdiagnosis in LDCT screening of LC [44]. In particular, the cumulative incidence rate of LC after 13 years of follow-up in the ITALUNG control arm was lower than in the active arm (RR: 0.89; 95% CI: 0.67–1.18).

The crucial role of follow-up length was confirmed by comparison of excess incidence and overdiagnosis estimates in two subsequent analyses in NLST. In fact, excess incidence in the active arm based on a follow-up of five years was 18.5% [70], whereas after a follow-up of 11.3 years the overall overdiagnosis estimate in the same arm was 3.1% [15].

### 2.7. Smoking-Related Comorbidities

Aging subjects with relevant smoking history have an increased risk of LC but also of additional smoking-related comorbidities. These mainly include CVD and respiratory diseases, especially chronic obstructive pulmonary disease (COPD) and interstitial lung disease (ILD). While these comorbidities can be assessed independently from LDCT [49,71,72,73], certainly, despite the low-dose acquisition technique, the screening chest CT itself allows post-test assessment of variables closely related to CVD, COPD, or ILD.

These smoking-related features in LDCT include CAC and calcification of the aortic valve, pulmonary emphysema, and increased thickness of the airways-wall related to chronic bronchitis (both underlying COPD), and parenchymal changes related to ILD. These collateral findings can be distinguished from findings unrelated to the smoking habit, more properly labelled “incidental findings”, that rarely have prognostic implications, but can require specific additional diagnostic workup [60].

In the wave of the special interest of our group in the assessment of COPD with CT or LDCT, clinically and in phantoms [74,75,76], in ITALUNG, we specifically investigated pulmonary emphysema. In particular, by applying lung densitometry, which is a more objective tool as compared to visual assessment [77,78], in subjects undergoing screening LDCT we assessed emphysema frequency and distribution (prevalence) [46,52], progression over time [47,48], and relevance in terms of long-term mortality [52]. 

Pulmonary emphysema was observed in about one-third of the ITALUNG participants [46,52] and was moderate or severe in 17% [52]. It infrequently and mildly progressed over time [48]. However, when moderate or severe at baseline LDCT, it was significantly associated with long-term overall and CVD mortality after adjustment for age, sex, smoking history, and CAC [52].

In a recent study, the densitometry evaluation of emphysema was compared with the quantification of diffuse lung damage using the CALIPER texture analysis [51]. Both methods were concordant in demonstrating lung changes related to smoking habits and their changes over time.

CAC represent another important comorbidity in smokers and former smokers undergoing screening LDCT. We evaluated in the whole cohort of subjects of the ITALUNG the extent of CAC in baseline LDCT using a reproducible and fast visual score which overcomes the difficulty of merging LDCT examinations obtained with several acquisition techniques and without cardiac gating in different CT scanners [50]. The distribution of the CAC at baseline LDCT and their predictive value when moderate or severe concerning long-term overall and CVD mortality in ITALUNG were in line with prior studies in which CAC were evaluated in LDCT examinations obtained using a single CT scanner [79,80]. 

### 2.8. Smoking Cessation

Along with the letter of proposal to participate in the ITALUNG, all randomized subjects received written information and access to a free smoking-cessation program (SCP). The SCP was available at the local smoking-cessation centres of the district of Florence, Pisa, and Pistoia. However, a more structured smoking intervention was performed at Pisa, where both the screening and the smoking-cessation centres are run by the same dedicated team of pneumologists. The SCP was based on individual physician-administered counselling and pharmacotherapy, with six visits in the first 3–5 months after a baseline evaluation and follow-up at 6 and 12 months [53].

Among ITALUNG participants who completed both baseline and four-year follow-up LDCT, higher quitting (20.8% vs. 16.7%, *p* = 0.029) and lower relapse (6.41% vs. 7.56%, *p* = 0.50) rates were observed in the active screening as compared to the usual-care control group, consistently with reports from other lung cancer-screening experiences. Quitting smoking was significantly associated with male gender, lower pack-years, and having pulmonary nodules at baseline LDCT. Maximal effect on quitting outcome was observed in participants in the SCP. As a novelty from the ITALUNG experience, it is noteworthy that the smokers who underwent the SCP at the Pisa centre showed higher CO-exhaled validated quitting rates at 12-month follow-up than matched controls from the general population who spontaneously entered the same SCP, in the same period, at the same centre. Thus, participating in a lung cancer-screening, such as the ITALUNG, and a SCP seems to effectively reinforce quitting smoking [53].

### 2.9. Radiations Exposure

One additional harm of LDCT screening of LC is exposure to the cancerogenic risk of ionizing radiations [81]. We anticipated the risk/benefit ratio of repeated annual LDCT over four years of ITALUNG considering different acquisition techniques and expected benefit in terms of decreased LC mortality [54]. Notably, for a LC mortality decrease of 20–30%, as reported in most trials, including our own, and acquisition techniques delivering less than 1 mSv, the potentially fatal cancers associated with radiation exposure were 0.11 per 1000 subjects for multi-detector CT scanners, which is about 10–100 times lower than the number of expected lives saved by screening in current smokers. 

After the end of the ITALUNG trial, we computed the cumulative mean effective dose delivered over four years to the single subject of the active arm that resulted between 6.2 and 6.8 mSv comprising four annual LDCT, accounting for 77.4% of the overall dose, as well as additional follow-up LDCT, FDG-PET examinations, and CT-guided fine-needle aspiration or core biopsy, accounting for the remainder dose [55]. By assuming the risk coefficients for stochastic effects after exposure to low-dose radiations indicated by the international and national agencies, the mean number of radiation-induced cancers in subjects undergoing LDCT in ITALUNG ranged between 0.12 and 0.33 per 1000 subjects.

Similar estimates, namely an additional risk of induced major cancers of 0.05%, were calculated in the COSMOS trial in Italy considering 10 years of active screening [82].

### 2.10. Biomarkers

For a long time, blood or sputum biomarkers have been investigated for LC screening with varying results [4,83]. In ITALUNG, samples of blood and sputum were collected before baseline LDCT and at recall for further assessment in 96% of the subjects of the active arm [57] with the aim to evaluate selected biomarkers as screening tools in combination with LDCT.

In a first study, we compared the performance of a grid of molecular genetic markers in blood and sputum, including allelic imbalance (loss of heterozygosity and microsatellite instability), free circulating DNA (fcDNA), K-ras mutations, and P53 mutation with respect to screen-detected LC diagnosed within the first year after baseline LDCT, positive baseline LDCT but no LC (benign nodules implying recall), and negative baseline LDCT [57]. Allelic imbalance in sputum or plasma was significantly more common in subjects with positive LDCT (benign nodule or LC) than in subjects with negative LDCT, whereas increased plasma fcDNA and K-ras mutations were almost exclusively observed in subjects with LC. 

In a second study, the biomarkers could be evaluated in additional 18 screen-detected LC and 2 interval cancers and in a larger sample of subjects who have completed the four LDCT screening rounds [58]. We assessed whether qualifying as positive any case with, at least, one abnormality among increased plasma cfDNA, loss of heterozygosity, and microsatellite instability, would increase the overall performance of the ITALUNG biomarker panel concerning diagnosis of LC. According to this definition, 94% of the LC diagnosed within one year of baseline LDCT were positive as well as 66% of LC diagnosed subsequently. Moreover, a simulation study indicated that a multimodal (LDCT plus IBP) approach could improve the efficiency of baseline screening and decrease the number of LDCT. 

## 3. Open Questions

Despite the now long history of LC screening with LDCT, several issues are still to be addressed (Table 2).

### 3.1. Design

After RCTs have established the validity of LDCT to screen for LC, it is unethical not to offer LDCT screening to adult or elderly subjects with significant smoking history with the exception of those who have quit smoking for many years and those unfit for thoracic surgery.

Today, how often and for how long to screen with LDCT are the open questions. Following a few preliminary studies [20,84], a large trial in Europe investigating the impact of annual vs. biennial LDCT on screening efficacy was launched in 2022 [85]. 

### 3.2. Recruitment

The COVID-19 epidemic has considerably hindered the accrual of LDCT screening, but only 17% of the target population adhered to LC screening in a US survey [86].

Although it is conceivable that the subject’s characteristics in terms of risk and comorbidities are not identical in opportunistic (self-referred) vs. population-based (organized) screening, with lower risk and generally better health conditions in subjects self-referring for a screening intervention [87], comparative data are being collected, for instance, in the CCM study in Italy [60], but are not yet available on this crucial issue. 

Consensus has been reached on the necessity of offering free access to SCP in subjects invited to LC screening with LDCT [27,85]. In fact, participation in a SCP is associated with a significant decrease in all-cause mortality in subjects attending LDCT screening [88,89]. However, the adhesion to SCP is variable [53], and the best SCP offering has not been established.

### 3.3. Structure

Several structures for screening LC with LDCT have been tested without a comparison in terms of efficiency and cost/benefit analyses. They include single-centre screening providing execution of LDCT, management of suspicious nodules and therapy [13,16,18,20], and multicentre screening with either peripheral LDCT reading and diagnostic workup [14,38] or centralized LDCT reading and peripheral diagnostic workup [85,90]. Each choice has advantages and disadvantages in terms of costs, expertise in LDCT reading, specific management of suspicious nodules, and subject’s discomfort related to traveling. 

### 3.4. Nodule Detection and Measurements

After an early phase in which the definition of the LDCT test result based on nodule size was variable from one study to another [12,14,90], Lung-RADS classification comprising both diameter and volume–size classes established the thresholds and terminology concerning nodule size measurements, as well as management recommendations [https://www.acr.org/-/media/ACR/Files/RADS/Lung-RADS/LungRADSAssessmentCategoriesv1-1.pdf?la=en, (accessed on 3 January 2023)]. This represents a distinct advantage for comparison of studies. Recently, in the Lung-RADS^®^ v2022, cystic lung lesions possibly related to LCCA and endobronchial abnormalities have also been incorporated. 

Computer-assisted diagnosis (CAD) systems have a considerable impact on reading the screening LDCT since they allow easier and faster detection of lung nodules, saving human resources, time, and costs [60]. However, experience in LC screening with LDCT is still limited [90,91]. 

Differently, a relatively large number of data are available concerning the use of software for the estimation of the volumes of lung nodules detected in screening LDCT [59,92]. Besides the persisting difficulties in the segmentation of some nodules and in volume estimate, it has been pointed out that some variability exists among different software and different releases of the same software [93]. Moreover, the measurement of the volume of non-solid nodules or non-solid components of mixed (part-solid) nodules is deemed unreliable [59]. 

The low frequency of malignancy among the many LDCT-detected nodules has stimulated the integration of individual risk factors and LDCT features to predict malignancy of a given nodule in a single subject with computation of probability risk scores [94,95]. Although the one developed at Brock University (Canada) considering nodule site, size, density, presence of spiculation, and beyond (familial history of LC, number of nodules, and presence of pulmonary emphysema), produced the best-performing score for prevalent malignant nodules in a validation study [95], its performance for incident malignant nodules was less satisfactory and it is conceivable that a dedicated probability-risk score for incident nodules is needed.

### 3.5. Results of LDCT

As in any screening intervention, the rate of positive LDCT tests implying a variety of management options is critical for the cost/effectiveness and, ultimately, sustainability of LC screening. Adhesion to Lung-RADS classification, especially with adoption of volumetric size measurements, is expected to contain the rate of positive LDCT tests at baseline and annual repeat LDCT. Similarly, a percentage of surgery for benign pathology below 10% is advocated [59]. While, in general, false positive tests are associated with distress and anxiety, on the one hand, unnecessary LDCT follow-up and FDG-PET examinations imply increased costs and cancerogenic risk deriving from ionizing radiations [55] and, on the other hand, unnecessary workup with CT-guided biopsies, bronchoscopy, and VATS is associated with the costs and harms of these invasive procedures.

False negative LDCT tests have been estimated in up to 15% of LC diagnosed in screening [96]. They have different sources that include inadequate radiological evaluation of the LDCT test, with detection or interpretation errors, nodule-management protocols, or management decisions in multidisciplinary sessions [30,96,97,98] and a screening interval exceeding two years [84]. The false negative rates must be taken as low as possible and represent a valuable metric for quality assurance in view of organized/population-based LC screening.

### 3.6. Outcomes 

Admittedly, the decrease in LC mortality associated with LDCT screening is mild–moderate, and there is wide room for improving the efficacy of LC screening in terms of mortality reduction (life years gained) while containing/reducing its harms [99]. 

Several potential actions can improve the efficiency of LC screening. By selecting higher-risk subjects identified with age or smoking history or ad hoc questionnaires, one may expect to increase the yield of screen-detected LC. However, unexpectedly, the people with a greater smoking burden are not those with the larger benefit of LC screening. This is due, on the one hand, to the higher incidence in these subjects of more aggressive and less curable LC histotypes as small-cell carcinoma and squamous-cell carcinoma [73,100,101] and, on the other hand, to the effect of comorbidities as competing causes of death, especially CVD, which substantially decrease the years of life that can be potentially gained in subjects with screening-detected LC [50,52,73,102,103]. 

Some studies emphasized the risk of overdiagnosis in LDCT screening, especially for broncho-alveolar carcinoma (BAC) appearing as non-solid or part-solid nodules [15,104]. Although watchful waiting has been recommended to avoid overtreatment [105,106], the optimal management of these indolent LC has not been established [107].

### 3.7. Smoking-Related Comorbidities

The correlation between pulmonary emphysema, COPD, and LC risk is established [108], although its determinants are unclear [109]. However, the presence or quantification of pulmonary emphysema in LDCT examinations is potentially relevant in establishing the individual risk of developing LC or the probability of malignancy of a lung nodule [94].

Moreover, the weight of smoking-related comorbidities in terms of CVD and respiratory disease in, ultimately, determining the efficacy of LDCT screening and in promoting its personalization has been recognized [103,110,111,112].

Incorporation in overall prognostic models of quantitative or semiquantitative CT features related to smoking-related comorbidities, including CAC, aortic valve calcifications, and pulmonary emphysema, has just been initiated [52,113] but requires further validations.

Great interest and expectations have raised the automatic assessment of comorbidities in subjects undergoing LC screening with LDCT with software already available for estimation of CV risk based on the presence, distribution, and severity of vascular calcifications [114] for pulmonary emphysema and interstitial lung disease quantification [51,75], for airways abnormalities underlying chronic bronchitis and COPD [115], and for other CT variables of potential interest, including bone, liver, and muscle density [116]. However, combination of this wealth of information in a balanced and efficient instrument also incorporating other risk variables appears a reasonable, but not yet at hand, goal.

### 3.8. Smoking Cessation

Lung cancer screening should be not considered a substitute for smoking cessation and smoking cessation is an essential part of the protocols in both research and clinical settings of LC screening [62,85,117]. The US Preventive Services Task Force Recommendation Statement (USPSTF) has made recommendations on behavioural and pharmacotherapy intervention for tobacco smoking cessation in screening for LC [27]. However, the optimal treatment type, timing of intervention, and content of communication, including the incorporation of CT results, to favour quitting smoking alongside participation in lung cancer screening are yet to be ascertained [118].

To determine how best to integrate SCP in the lung screening setting, the National Cancer Institute initiated the Smoking Cessation at Lung Examination (SCALE) collaboration [119,120] that is comprised of eight clinical trials. To date, some of these studies have provided evidence for the integration of SCP in the lung screening context. Offering multiple accrual methods and at multiple points during the screening regimen may help to engage current smokers, and, by providing pharmacotherapy options, to promote enrolment [121]. As expected, treatment engagement and retention reflect demographic, clinical, and psychological characteristics (e.g., number of cigarettes smoked per day, education, worry about LC, and screening results) [122].

Recently, it has been shown that the combination of immediate cessation and pharmacotherapy support is an effective method for quitting smoking and can be delivered within a screening context [123,124]. Such an approach is in line with the ITALUNG experience.

### 3.9. Radiations Exposure

Despite the persistent lack of cancer incidence studies in subjects recruited in LC screening studies [56], it is conceivable to assume that the radiation exposure and cancer risk induction from low-dose CT is non-negligible, but acceptable in light of the substantial mortality reduction associated with screening. 

Nevertheless, also considering the 30 annual LDCT examinations recommended by the USPSTF in a 50-year-old smoker initiating LC screening [27], several studies have investigated the capability of iterative algorithms to reconstruct the CT images while decreasing the radiation dose associated with screening CT below the 1 mSv, so-called ultra-low-dose CT (ULDCT) [125,126,127,128]. However, so far, ULDCT has not been fully validated for substituting LDCT for LC screening.

### 3.10. Biomarkers

The expected features for a really useful biomarker or biomarkers panel aim at meeting two main as-yet unmet clinical needs: (1) risk stratification to improve the selection of individuals undergoing screening; and (2) management of undetermined nodules detected by LDCT screening [83].

Although numerous studies have evaluated biomarkers as indicators of LC, so far, no screening study has included them as part of the protocol [129,130]. Nevertheless, the results of several ongoing studies are encouraging. MicroRNA signature shows promising accuracy in predicting lung cancer risk and in defining adequate screening intervals [131]. As smoking is associated with epigenetic modification, DNA methylation shows high diagnostic accuracy for detection of early-stage LC [132] and provide independent risk information to identify eligible smokers for screening [133]. Liquid biopsy represents a practical alternative source for investigating tumour-derived somatic alterations with a minimally invasive approach, including a variety of methodologies for circulating analytes. Plasma-circulating tumour DNA (ctDNA) is the most extensively studied and widely adopted alternative to tissue-tumour genotyping in solid tumours, first entering clinical practice for detection of EGFR mutations in non-small-cell lung cancer [134].

Subjects enrolled in trials evaluating LCDT represent the ideal population in which to study a combined bio instrumental approach for screening [135]. The ITALUNG biobank, containing biospecimens standardly collected at baseline and in the follow-up of non-calcified lung nodules, represents a source of high-quality samples, useful to generate accurate, precise, and reliable biomarkers studies for which international collaborations are ongoing. 

## 4. Artificial Intelligence and LC Screening 

Today, artificial intelligence (AI) is pervading every aspect of daily life and medicine. Its implementation is expected to solve some of the problems of LC screening that we have outlined above [136,137]. In particular, machine-learning can be used to approach processes requiring analysis of different numerical information, including prediction of the risk of lung cancer based on non-imaging variables [138] and of overall and cause-specific mortality in subjects undergoing screening based on assessment of emphysema and CAC in baseline LDCT [52,113,139]. On its turn, automatic analyses of LDCT images with deep-learning algorithms helps in detection of lung nodules [140,141,142], nodule characterization in terms of malignancy [143,144], quantification of vascular calcifications [114,145], assessment of diffuse lung abnormalities [146,147], and prediction of future LC risk without clinical or demographic data [148]. Moreover, it is anticipated that algorithms combining LDCT, clinical, and laboratory features might help to keep both false negatives and false positives very low in the context of generalised screening programs. 

## 5. Conclusions

The 20-plus years of experience in lung cancer screening with LDCT in the ITALUNG trial has allowed the accumulation of new scientific evidence about several features of early LC diagnosis and to complete a learning curve for radiologists and physicians. Implementation of AI promises to help solving persistent uncertainties about whom, how, and for how long to screen for LC among subjects with a smoking history.

## Figures and Tables

**Table 1:** Published scientific articles from the ITALUNG study group.

	Ref.
**Design and recruitment**	
Picozzi, G., et al. Screening of lung cancer with low dose spiral CT: results of a three year pilot study and design of the randomised controlled trial ‘‘Italung-CT’’. Radiol Med. 2005 Jan-Feb;109(1–2):17–26. PMID: 15729183	[30]
Lopes Pegna, A., et al. Design, recruitment and baseline results of the ITALUNG trial for lung cancer screening with low-dose CT. Lung Cancer. 2009 Apr;64(1):34–40. doi: 10.1016/j.lungcan.2008.07.003. PMID: 18723240	[21]
**Nodule detection and measurements**	
Bellotti, R., et al. A CAD system for nodule detection in low-dose lung CTs based on region growing and a new active contour model. Med Phys. 2007 Dec;34(12):4901–10. doi: 10.1118/1.2804720. PMID: 18196815	[31]
Golosio, B., et al. A novel multithreshold method for nodule detection in lung CT. Med Phys. 2009 Aug;36(8):3607–18. doi: 10.1118/1.3160107. PMID: 19746795	[32]
Picozzi, G., et al. Operator-dependent reproducibility of size measurements of small phantoms and lung nodules examined with low-dose thin-section computed tomography. Invest Radiol. 2006 Nov;41(11):831–9. doi: 10.1097/01.rli.0000242837.11436.6e. PMID: 17035874.	[33]
Diciotti, S., et al. 3-D segmentation algorithm of small lung nodules in spiral CT images. IEEE Trans Inf Technol Biomed. 2008 Jan;12(1):7–19. doi: 0.1109/TITB.2007.899504. PMID: 18270032	[34]
Diciotti, S., et al. The LoG characteristic scale: a consistent measurement of lung nodule size in CT imaging. IEEE Trans Med Imaging. 2010 Feb;29(2):397–409. doi: 10.1109/TMI.2009.2032542. PMID: 20129846	[35]
De Nunzio, G., et al. Automatic lung segmentation in CT images with accurate handling of the hilar region. J Digit Imaging. 2011 Feb;24(1):11–27. doi: 10.1007/s10278-009-9229-1. PMID: 19826872	[36]
Diciotti, S., et al. Automated segmentation refinement of small lung nodules in CT scans by local shape analysis. IEEE Trans Biomed Eng. 2011 Dec;58(12):3418–28. doi: 10.1109/TBME.2011.2167621. PMID: 21914567	[37]
**Results of LDCT**	
Lopes Pegna, A., et al. Four-year results of low-dose CT screening and nodule management in the ITALUNG trial. J Thorac Oncol. 2013 Jul;8(7):866–75. doi: 10.1097/JTO.0b013e31828f68d6. PMID: 23612465	[38]
Mascalchi, M., et al. Initial LDCT appearance of incident lung cancers in the ITALUNG trial. Eur J Radiol. 2014 Nov;83(11):2080–6. doi: 10.1016/j.ejrad.2014.07.019. PMID: 25174775	[39]
Mascalchi, M., Sali, L. Risk of Second Lung Cancer in ITALUNG LDCT Screening. J Thorac Oncol. 2018 Jun;13(6):e105-e106. doi: 10.1016/j.jtho.2018.02.027. PMID: 29793649	[40]
Mascalchi, M., et al. Screen-detected multiple primary lung cancers in the ITALUNG trial. J Thorac Dis. 2018 Feb;10(2):1058–1066. doi: 10.21037/jtd.2018.01.95. PMID: 29607181	[41]
**Outcomes**	
Mascalchi, M., et al. Does UKLS strategy increase the yield of screen-detected lung cancers? A comparison with ITALUNG.Thorax. 2016 Oct;71(10):950–1. doi: 10.1136/thoraxjnl-2016-208409. PMID: 27217521	[42]
Paci, E., et al. Mortality, survival and incidence rates in the ITALUNG randomised lung cancer screening trial. Thorax. 2017 Sep;72(9):825–831. doi: 10.1136/thoraxjnl-2016-209825. PMID: 28377492	[43]
Paci, E., et al. Prognostic selection and long-term survival analysis to assess overdiagnosis risk in lung cancer screening randomized trials. J Med Screen. 2021 Mar;28(1):39–47. doi: 10.1177/0969141320923030. PMID: 32437229	[44]
Puliti, D., et al. Gender effect in the ITALUNG screening trial. A comparison with UKLS and other trials. Lancet Reg Health Eur. 2022 Jan 1;13:100300. doi: 10.1016/j.lanepe.2021.100300. eCollection 2022 Feb. PMID: 35024679	[45]
** Smoking-related comorbidities**	
Camiciottoli, G., et al. Prevalence and correlates of pulmonary emphysema in smokers and former smokers. A densitometric study of participants in the ITALUNG trial. Eur Radiol. 2009 Jan;19(1):58–66. doi: 10.1007/s00330-008-1131-6. PMID: 18690451	[46]
Diciotti, S., et al. Defining the intra-subject variability of whole-lung CT densitometry in two lung cancer screening trials. Acad Radiol. 2011 Nov;18(11):1403–11. doi: 10.1016/j.acra.2011.08.001. PMID: 21971258	[47]
Mascalchi, M. et al. Changes in volume-corrected whole-lung density in smokers and former smokers during the ITALUNG screening trial. J Thorac Imaging. 2012 Jul;27(4):255–62. doi: 10.1097/RTI.0b013e3182541165. PMID: 22576761	[48]
Puliti, D., et al. Decreased cardiovascular mortality in the ITALUNG lung cancer screening trial: Analysis of underlying factors.Lung Cancer. 2019 Dec;138:72–78. doi: 10.1016/j.lungcan.2019.10.006. PMID: 31654837	[49]
Mascalchi, M., et al. Moderate-severe coronary calcification predicts long-term cardiovascular death in CT lung cancer screening: The ITALUNG trial. Eur J Radiol. 2021 Dec;145:110040. doi: 10.1016/j.ejrad.2021.110040. PMID: 34814037	[50]
Romei, C., et al. Quantitative texture-based analysis of pulmonary parenchymal features on chest CT: comparison with densitometric indices and short-term effect of changes in smoking habit. Eur Respir J. 2022 Oct 13;60(4):2102618. doi: 10.1183/13993003.02618–2021 PMID: 35604814	[51]
Mascalchi, M., et al. Pulmonary emphysema and coronary artery calcifications at baseline LDCT and long-term mortality in smokers and former smokers of the ITALUNG screening trial. Eur Radiol. 2023 May;33(5):3115–3123. doi: 10.1007/s00330-023-09504-4. PMID: 36854875.	[52]
**Smoking cessation**	
Pistelli, F., et al. Smoking Cessation in the ITALUNG Lung Cancer Screening: What Does "Teachable Moment" Mean? Nicotine Tob Res. 2020 Aug 24;22(9):1484–1491. doi: 10.1093/ntr/ntz148. PMID: 31504798	[53]
**Radiations Exposure**	
Mascalchi, M., et al. Risk-benefit analysis of X-ray exposure associated with lung cancer screening in the Italung-CT trial. AJR Am J Roentgenol. 2006 Aug;187(2):421–9. doi: 10.2214/AJR.05.0088. PMID: 16861547	[54]
Mascalchi, M., et al. Dose exposure in the ITALUNG trial of lung cancer screening with low-dose CT. Br J Radiol. 2012 Aug;85(1016):1134–9. doi: 10.1259/bjr/20711289. PMID: 21976631	[55]
Mascalchi, M., Sali, L. Lung cancer screening with low dose CT and radiation harm-from prediction models to cancer incidence data. Ann Transl Med. 2017 Sep;5(17):360. doi: 10.21037/atm.2017.06.41. PMID: 28936454	[56]
**Biomarkers**	
Carozzi, F.M., et al. Molecular profile in body fluids in subjects enrolled in a randomised trial for lung cancer screening: Perspectives of integrated strategies for early diagnosis. Lung Cancer. 2010 May;68(2):216–21. doi: 10.1016/j.lungcan.2009.06.015. PMID: 19646775	[57]
Carozzi, F.M., et al. Multimodal lung cancer screening using the ITALUNG biomarker panel and low dose computed tomography. Results of the ITALUNG biomarker study. Int J Cancer. 2017 Jul;141(1):94–101. doi: 10.1002/ijc.30727. PMID: 28387927	[58]
**Position papers**	
Oudkerk, M., et al. European position statement on lung cancer screening. Lancet Oncol. 2017 Dec;18(12):e754-e766. doi: 10.1016/S1470-2045(17)30861-6. PMID: 29208441.	[59]
Silva, M., et al. Low-dose CT for lung cancer screening: position paper from the Italian college of thoracic radiology. Radiol Med. 2022 May;127(5):543–559. doi: 10.1007/s11547-022-01471-y. PMID: 35306638.	[60]

**Table 2:** Unresolved issues in lung cancer screening with low-dose CT.

**a.** **Design**
Annual or biennial screening—other schemes
**b.** **Recruitment**
Population (organized) or opportunistic (self-referred) screening
**c.** **Structure**
Single center or multicenter with centralized or peripheral LDCT reading and management
**d.** **Nodule detection and measurements**
Implementation of CAD Improvement and validatation for volumetry of non-solid nodules or components Improvement of risk scores of malignancy for incident nodules
**e.** **Results of LDCT**
Containment of false positive and false negative tests
**f.** **Outcomes**
Enhance the decrease of LC mortality associated with LDCT screening
**g.** **Smoking-related comorbidities**
Quantification of smoking-related comorbidities with incorporation in personalized models of LC and mortality risk
**h.** **Smoking cessation**
Optimization of engagement in smoking cessation programs within lung cancer screening Optimization of type and timing of treatment (including content of communication and pharmacotherapy)
**i.** **Radiations exposure**
Validation of ultra low-dose computed tomography
**j.** **Biomarkers**
Prospective evaluation in combination with LDCT

## Data Availability

The data presented in this review articles can be retrieved from the references detailed below and from the www addresses indicated in the text.
